# Supplementary material for: Sources and preferences for nutrition information among older adults: A scoping review
Source: PLoS One. 2026 Feb 27;21(2):e0341015. doi: 10.1371/journal.pone.0341015 (PMC12948054; doi:10.1371/journal.pone.0341015)
Supplement: S1 File — (PPTX) [file pone.0341015.s001.pptx]

## Slide 1
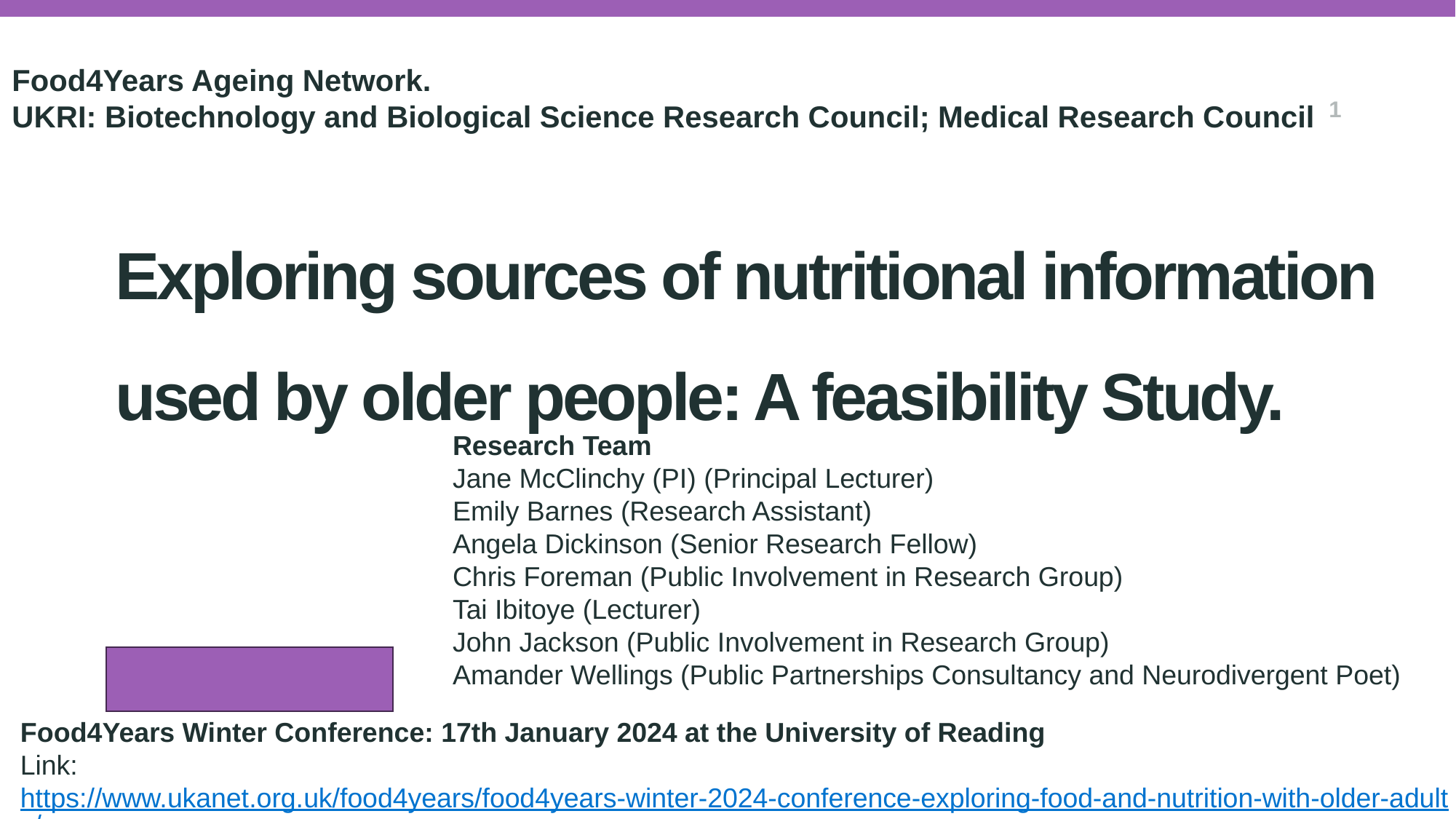

Food4Years Ageing Network.
UKRI: Biotechnology and Biological Science Research Council; Medical Research Council
1
# Exploring sources of nutritional information used by older people: A feasibility Study.
Research Team
Jane McClinchy (PI) (Principal Lecturer)​
Emily Barnes (Research Assistant)​
Angela Dickinson (Senior Research Fellow)
Chris Foreman (Public Involvement in Research Group)​
Tai Ibitoye (Lecturer)​
John Jackson (Public Involvement in Research Group)​
Amander Wellings (Public Partnerships Consultancy and Neurodivergent Poet)​
Food4Years Winter Conference: 17th January 2024 at the University of Reading
Link: https://www.ukanet.org.uk/food4years/food4years-winter-2024-conference-exploring-food-and-nutrition-with-older-adults/ Programme: https://www.ukanet.org.uk/food4years/programme-2/

## Slide 2
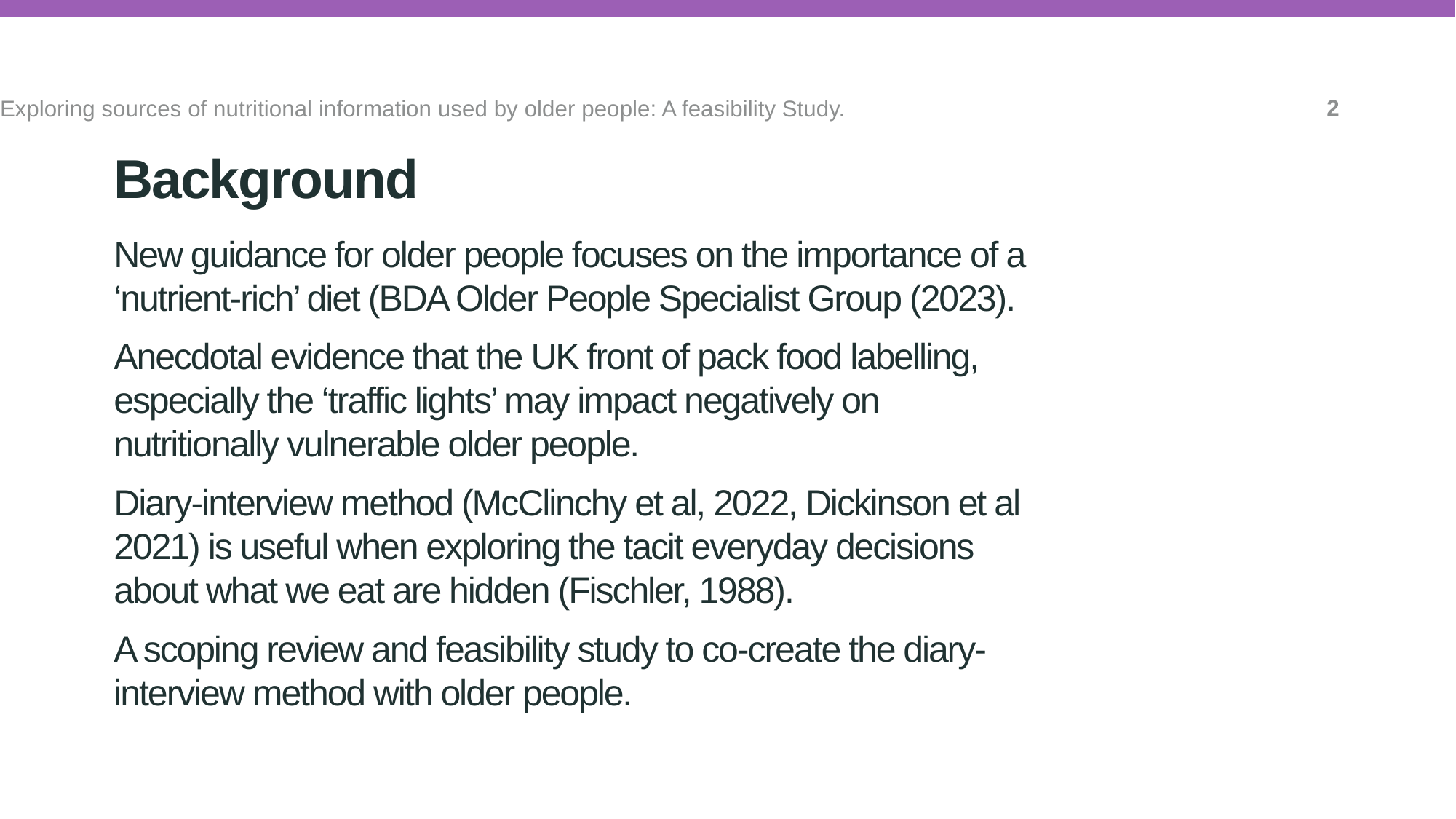

2
Exploring sources of nutritional information used by older people: A feasibility Study.
# Background
New guidance for older people focuses on the importance of a ‘nutrient-rich’ diet (BDA Older People Specialist Group (2023).
Anecdotal evidence that the UK front of pack food labelling, especially the ‘traffic lights’ may impact negatively on nutritionally vulnerable older people.
Diary-interview method (McClinchy et al, 2022, Dickinson et al 2021) is useful when exploring the tacit everyday decisions about what we eat are hidden (Fischler, 1988).
A scoping review and feasibility study to co-create the diary-interview method with older people.

## Slide 3
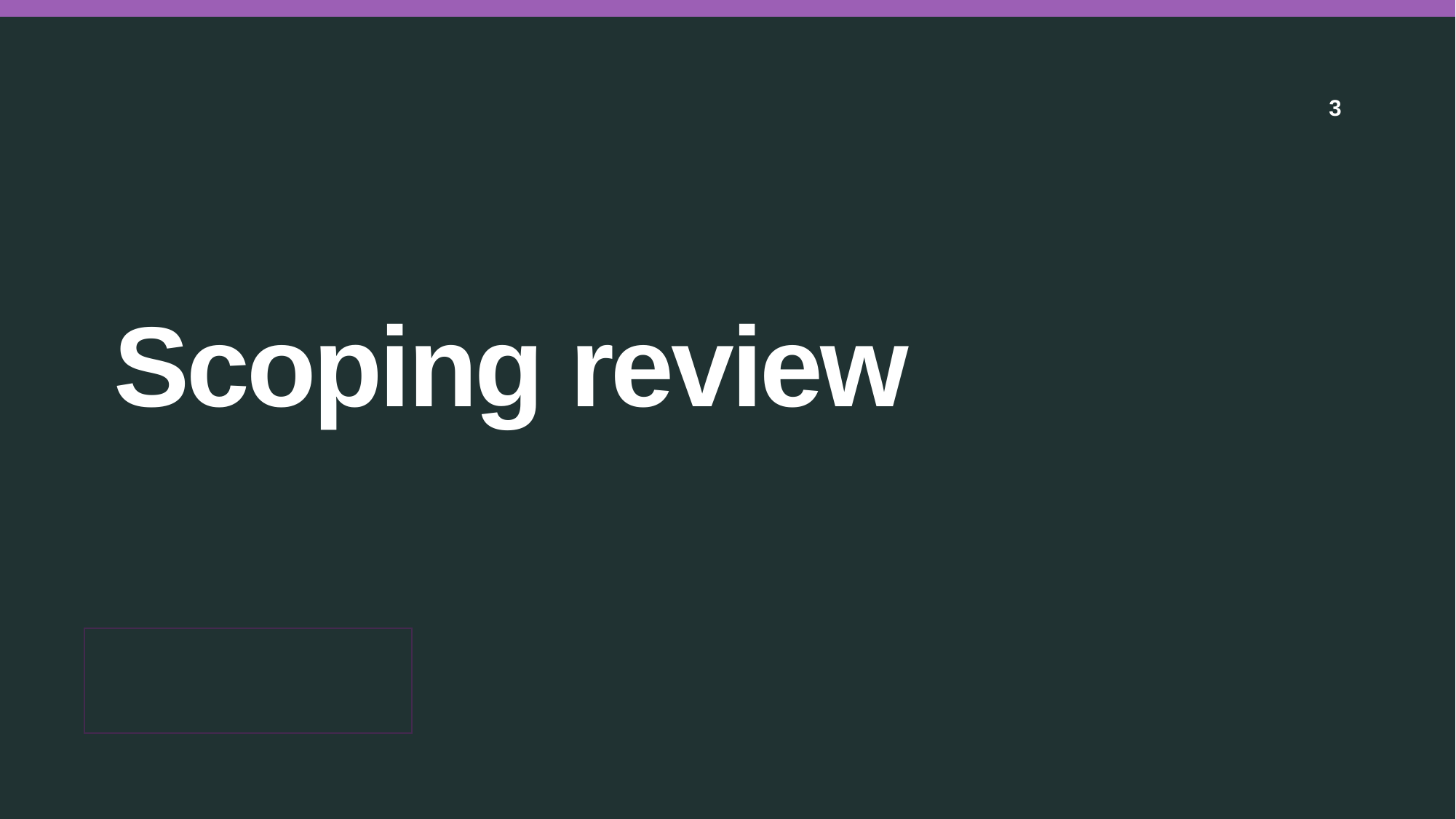

3
# Scoping review

## Slide 4
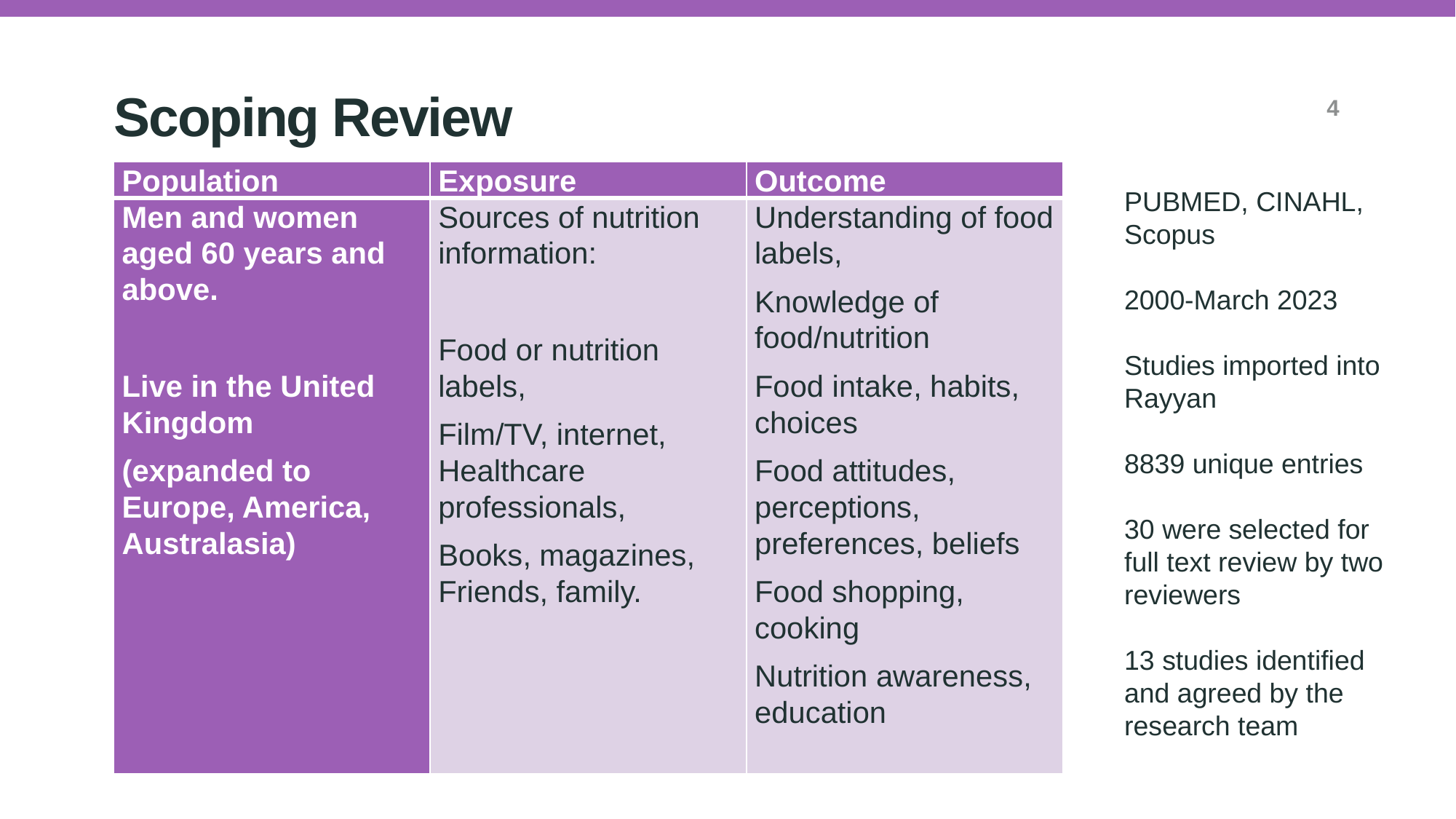

# Scoping Review
4
| Population | Exposure | Outcome |
| --- | --- | --- |
| Men and women aged 60 years and above.   Live in the United Kingdom (expanded to Europe, America, Australasia) | Sources of nutrition information:   Food or nutrition labels, Film/TV, internet, Healthcare professionals, Books, magazines, Friends, family. | Understanding of food labels, Knowledge of food/nutrition Food intake, habits, choices Food attitudes, perceptions, preferences, beliefs Food shopping, cooking Nutrition awareness, education |
PUBMED, CINAHL, Scopus
2000-March 2023
Studies imported into Rayyan
8839 unique entries
30 were selected for full text review by two reviewers
13 studies identified and agreed by the research team

## Slide 5
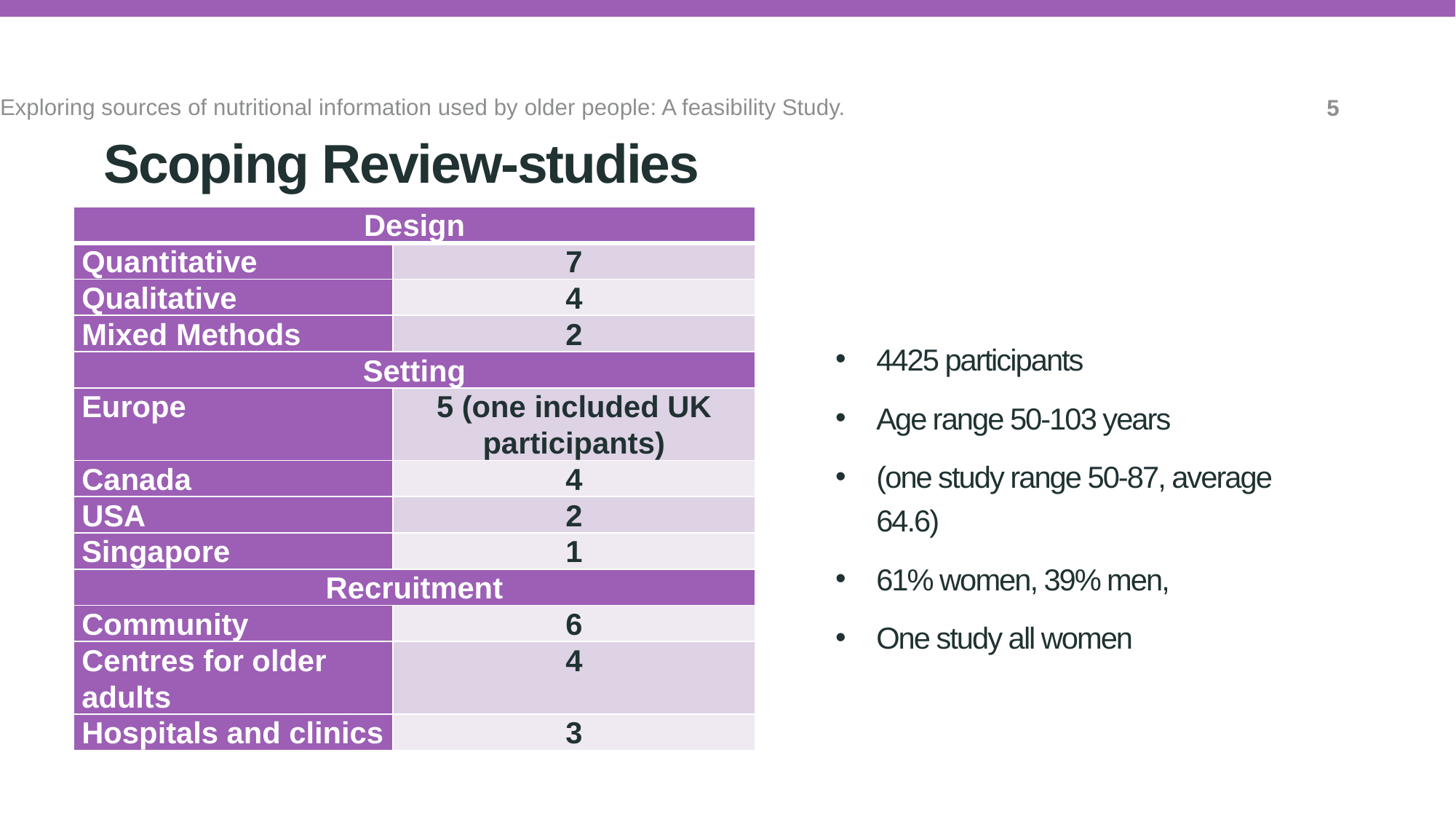

Exploring sources of nutritional information used by older people: A feasibility Study.
5
# Scoping Review-studies
| Design | |
| --- | --- |
| Quantitative | 7 |
| Qualitative | 4 |
| Mixed Methods | 2 |
| Setting | |
| Europe | 5 (one included UK participants) |
| Canada | 4 |
| USA | 2 |
| Singapore | 1 |
| Recruitment | |
| Community | 6 |
| Centres for older adults | 4 |
| Hospitals and clinics | 3 |
4425 participants
Age range 50-103 years
(one study range 50-87, average 64.6)
61% women, 39% men,
One study all women

## Slide 6
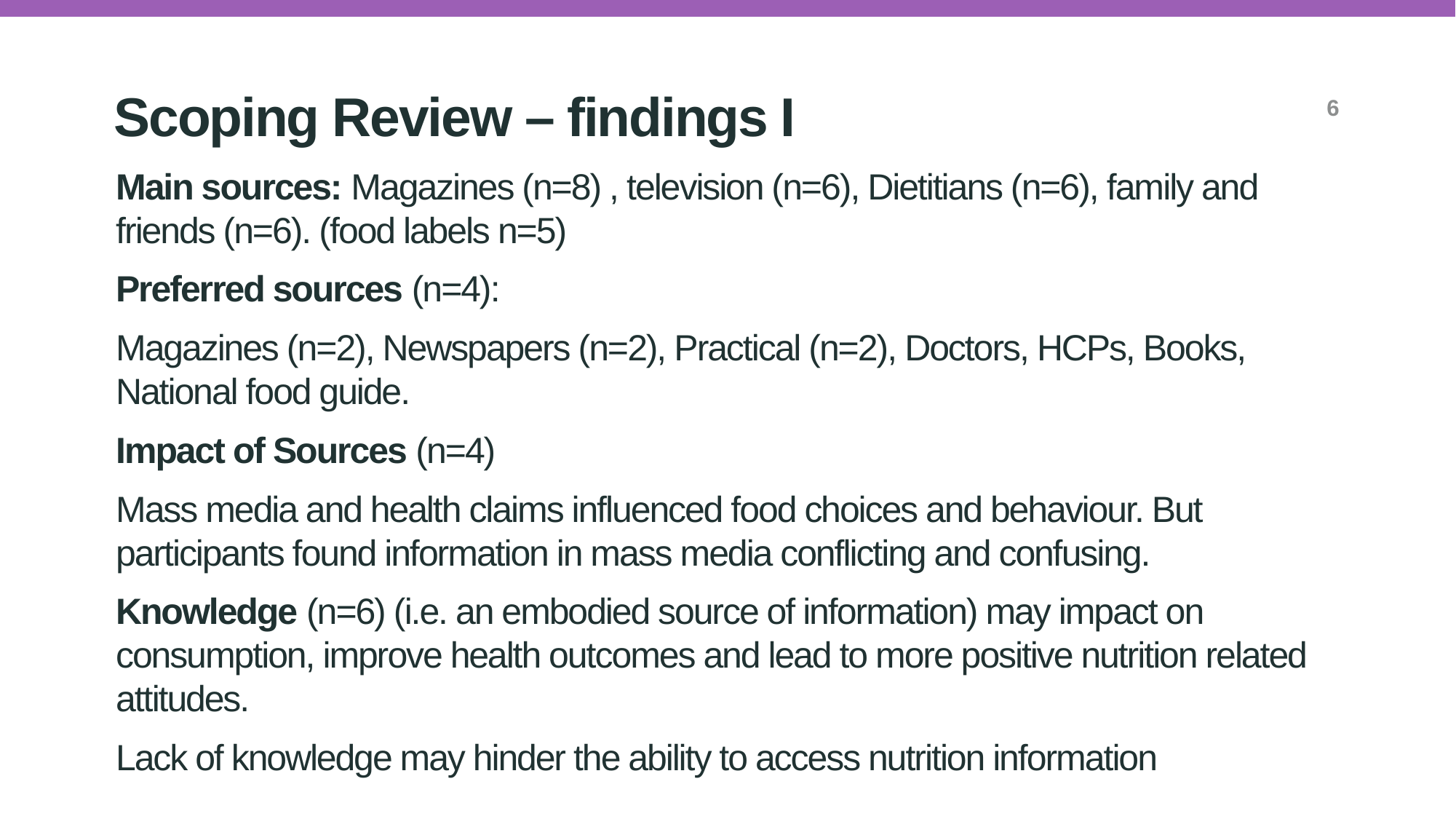

# Scoping Review – findings I
6
Main sources: Magazines (n=8) , television (n=6), Dietitians (n=6), family and friends (n=6). (food labels n=5)
Preferred sources (n=4):
Magazines (n=2), Newspapers (n=2), Practical (n=2), Doctors, HCPs, Books, National food guide.
Impact of Sources (n=4)
Mass media and health claims influenced food choices and behaviour. But participants found information in mass media conflicting and confusing.
Knowledge (n=6) (i.e. an embodied source of information) may impact on consumption, improve health outcomes and lead to more positive nutrition related attitudes.
Lack of knowledge may hinder the ability to access nutrition information

## Slide 7
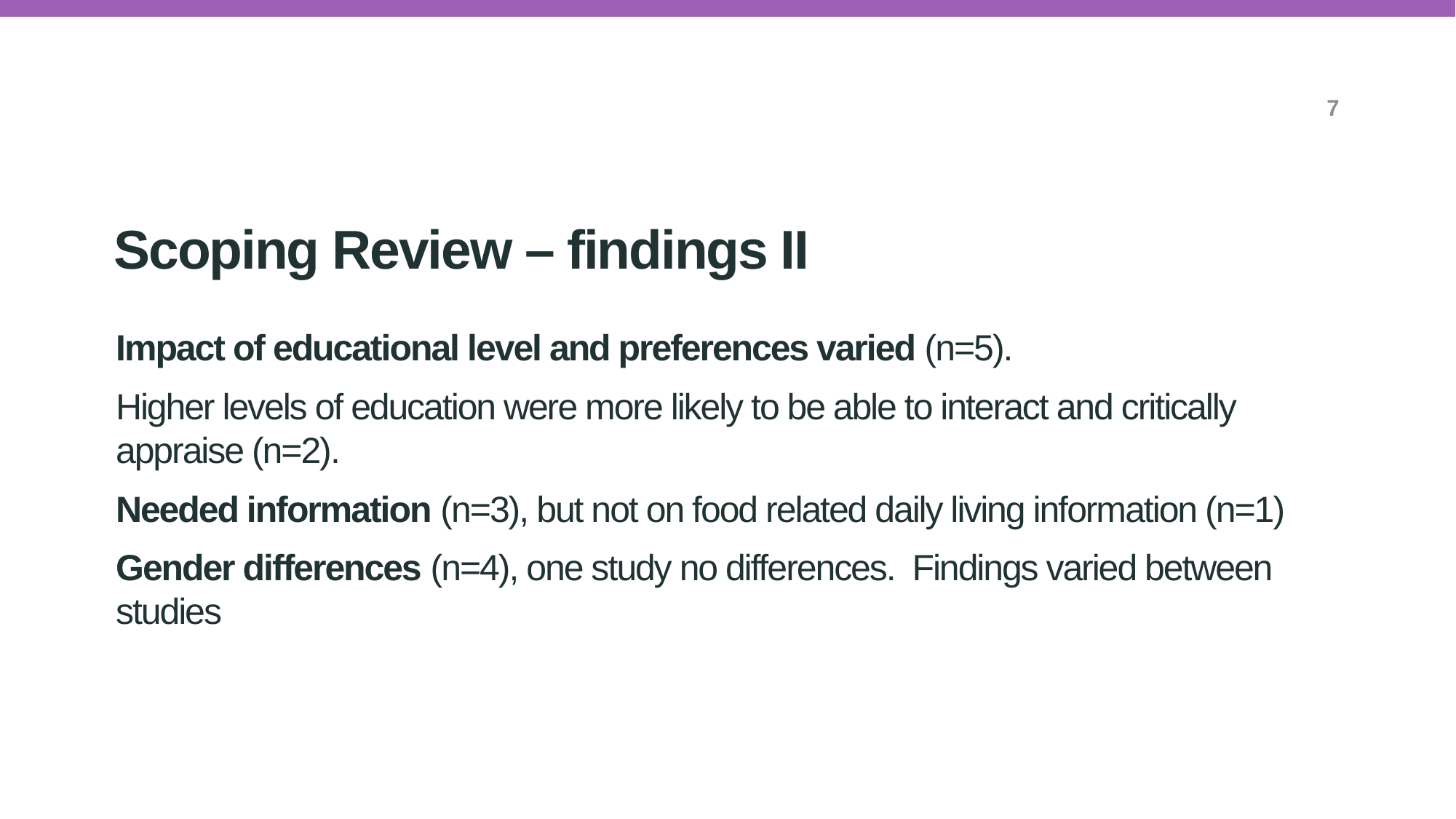

7
# Scoping Review – findings II
Impact of educational level and preferences varied (n=5).
Higher levels of education were more likely to be able to interact and critically appraise (n=2).
Needed information (n=3), but not on food related daily living information (n=1)
Gender differences (n=4), one study no differences. Findings varied between studies

## Slide 8
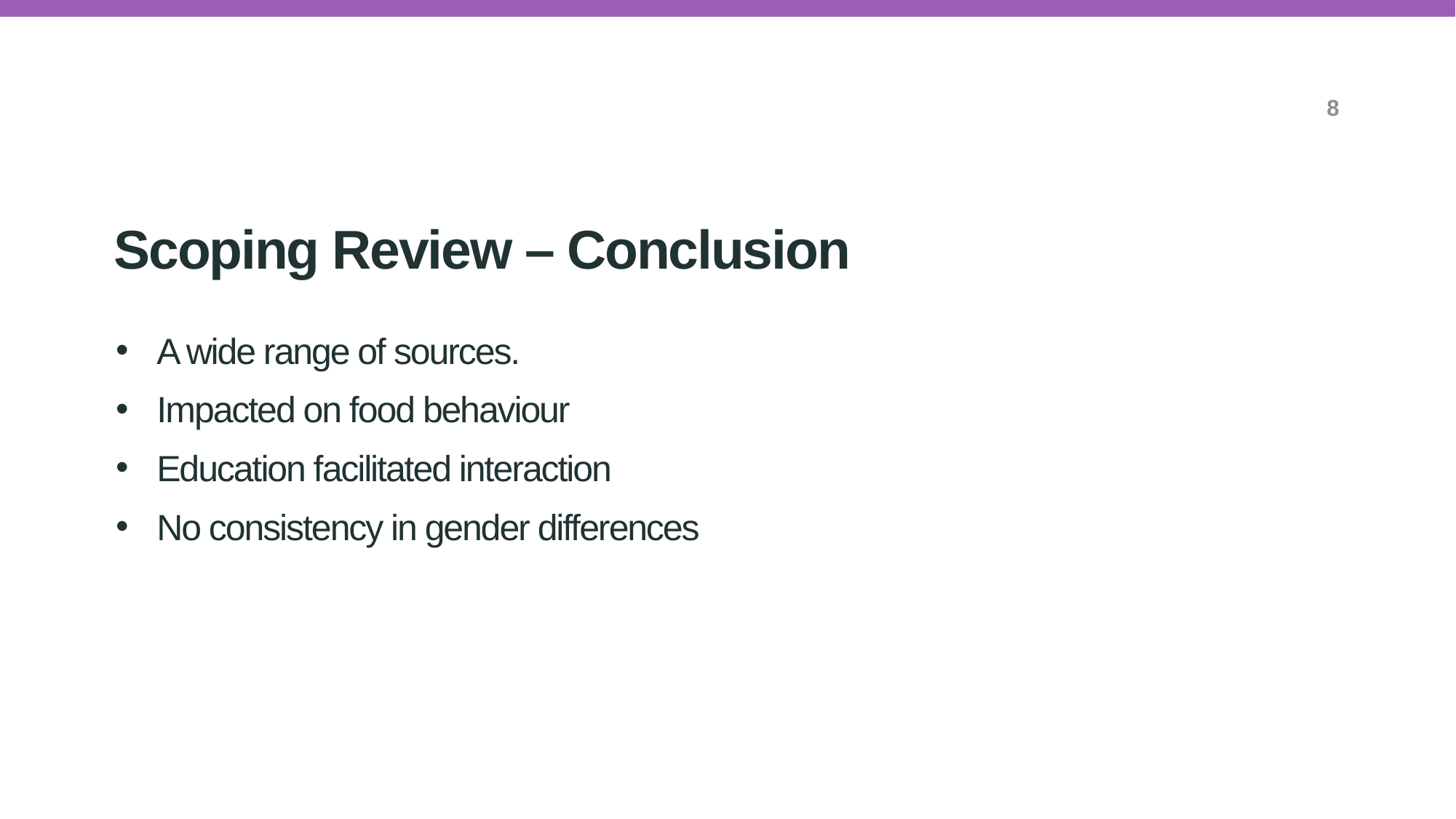

8
# Scoping Review – Conclusion
A wide range of sources.
Impacted on food behaviour
Education facilitated interaction
No consistency in gender differences

## Slide 9
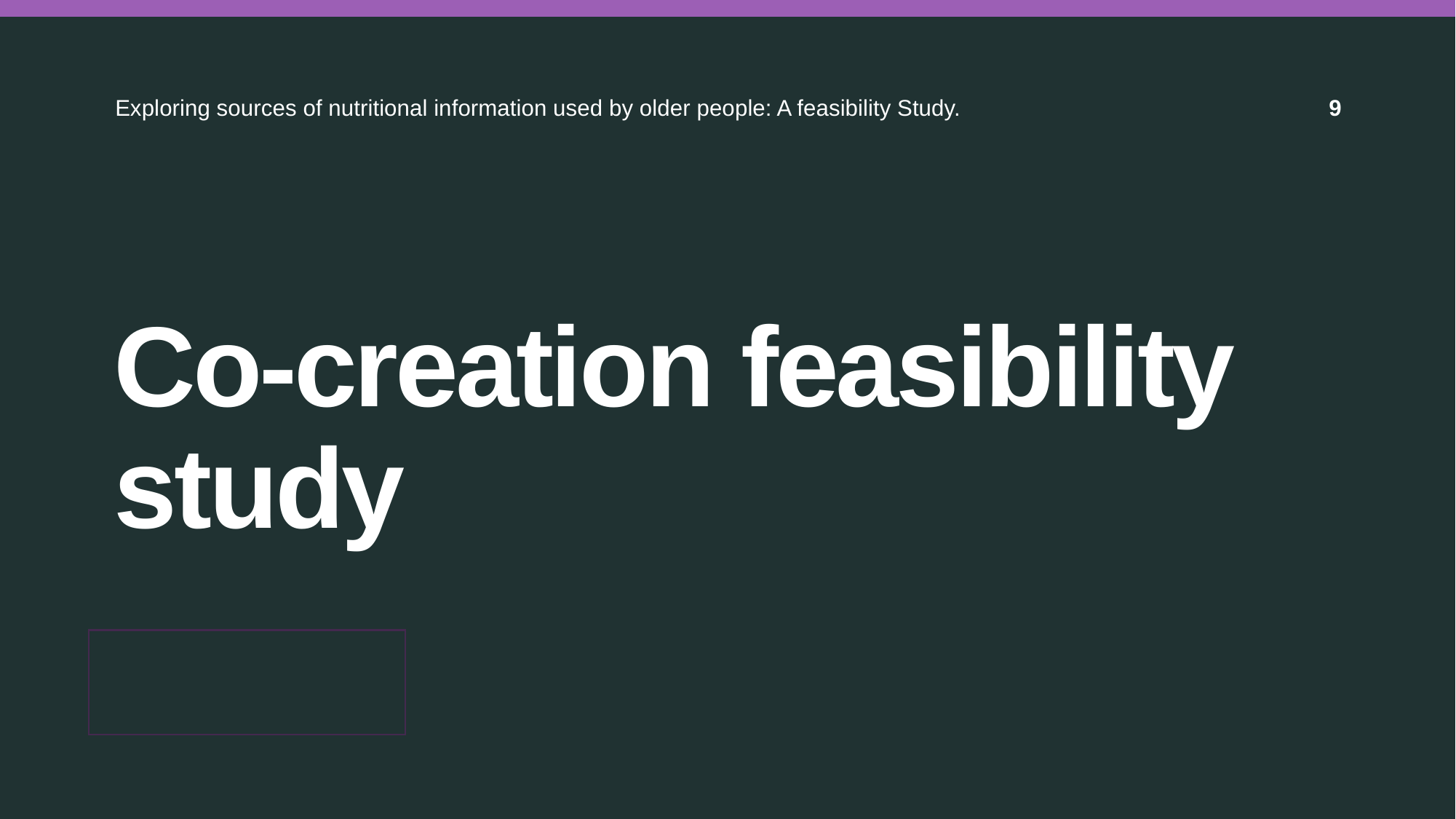

Exploring sources of nutritional information used by older people: A feasibility Study.
9
# Co-creation feasibility study

## Slide 10
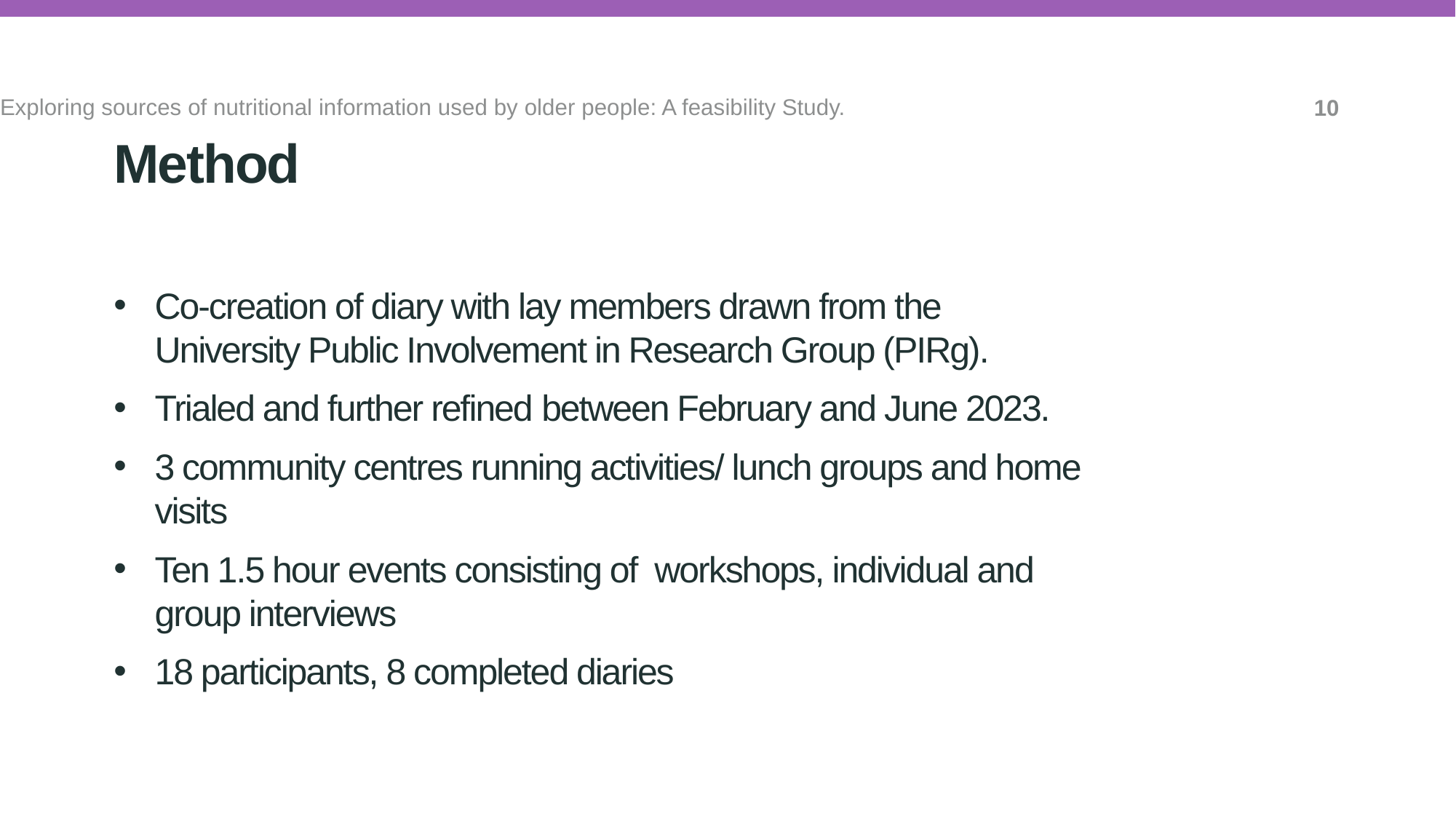

Exploring sources of nutritional information used by older people: A feasibility Study.
10
# Method
Co-creation of diary with lay members drawn from the University Public Involvement in Research Group (PIRg).
Trialed and further refined between February and June 2023.
3 community centres running activities/ lunch groups and home visits
Ten 1.5 hour events consisting of  workshops, individual and group interviews
18 participants, 8 completed diaries

## Slide 11
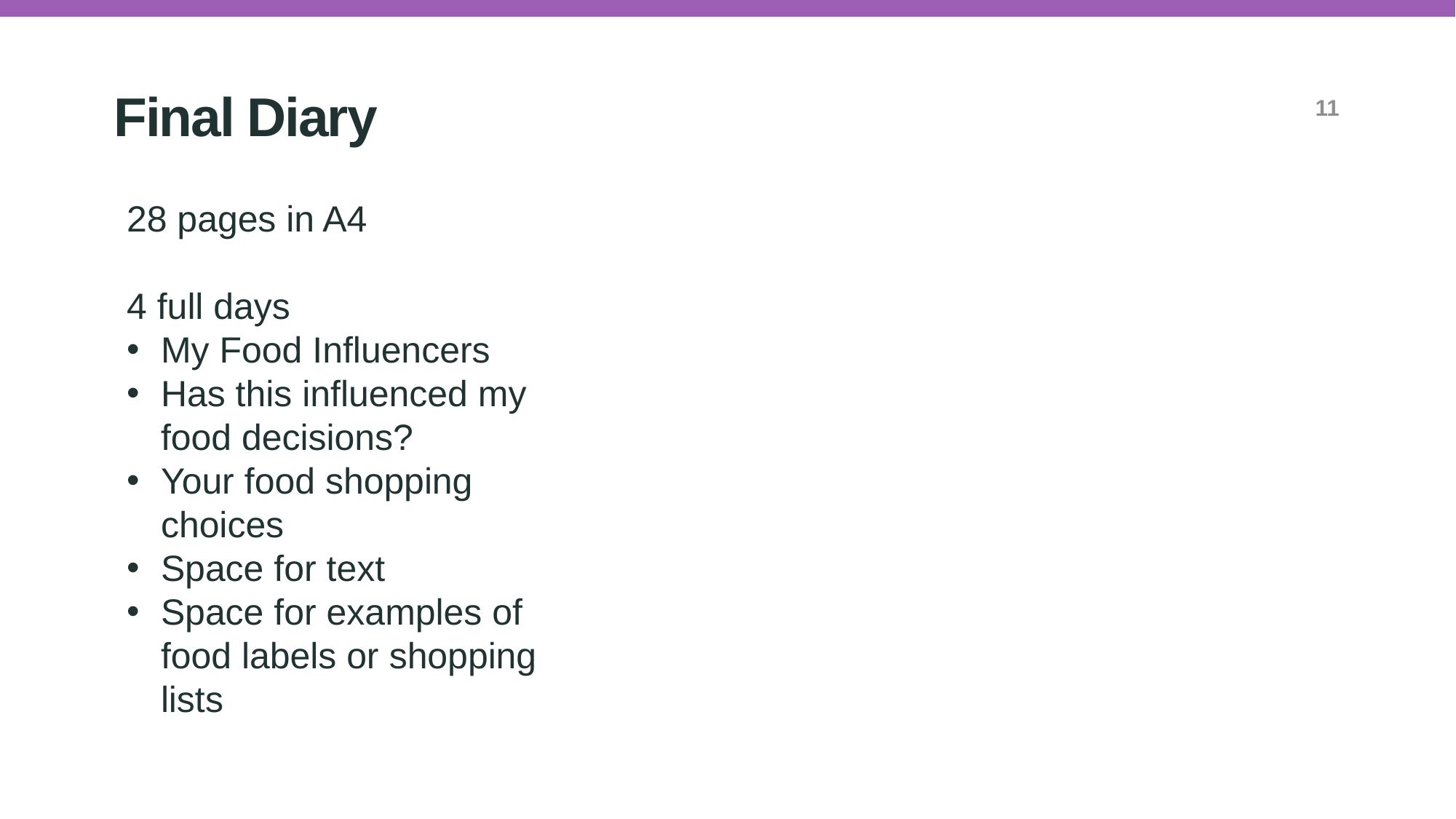

# Final Diary
11
28 pages in A4
4 full days
My Food Influencers
Has this influenced my food decisions?
Your food shopping choices
Space for text
Space for examples of food labels or shopping lists

## Slide 12
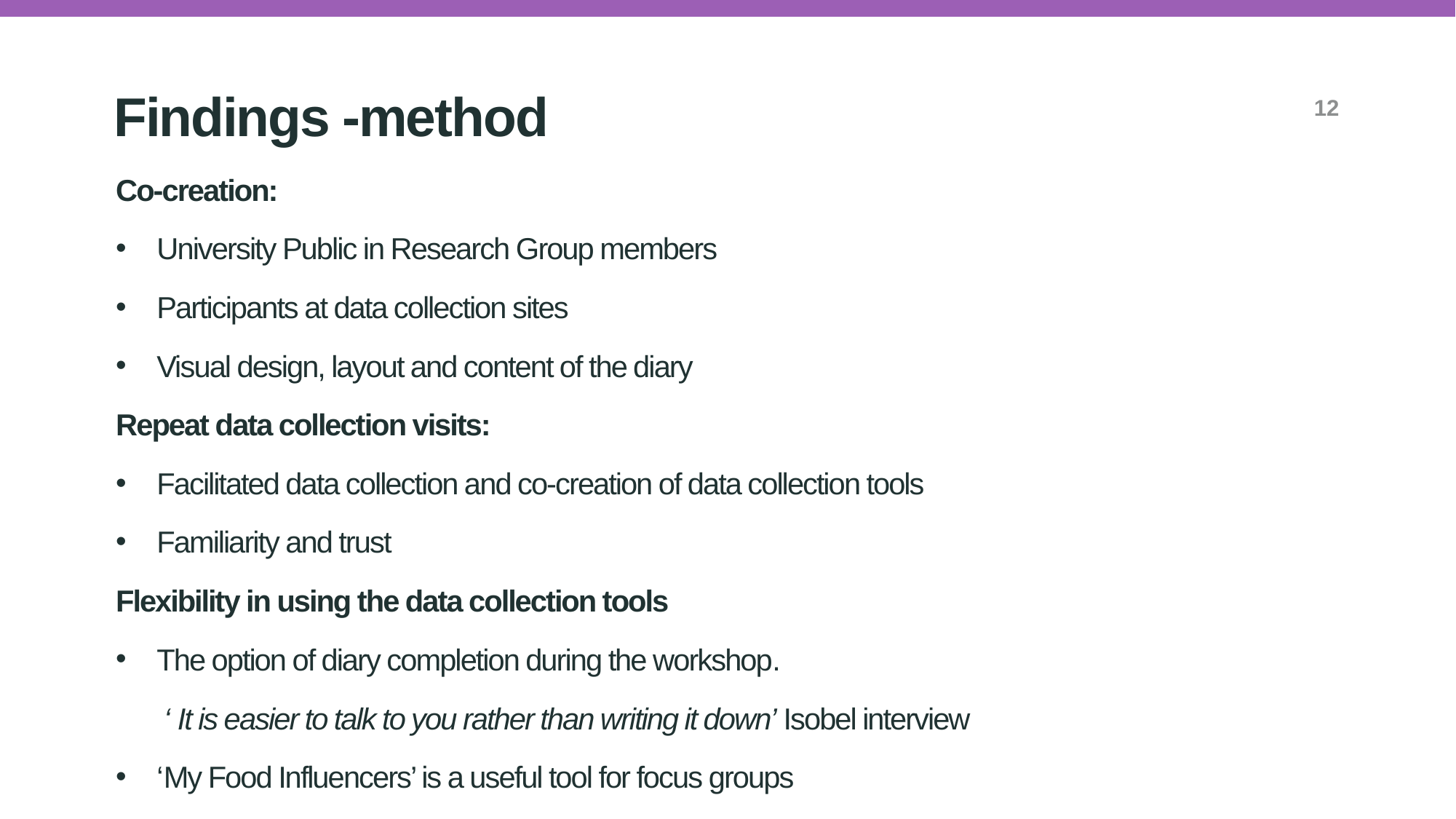

# Findings -method
12
Co-creation:
University Public in Research Group members
Participants at data collection sites
Visual design, layout and content of the diary
Repeat data collection visits:
Facilitated data collection and co-creation of data collection tools
Familiarity and trust
Flexibility in using the data collection tools
The option of diary completion during the workshop.
       ‘ It is easier to talk to you rather than writing it down’ Isobel interview
‘My Food Influencers’ is a useful tool for focus groups

## Slide 13
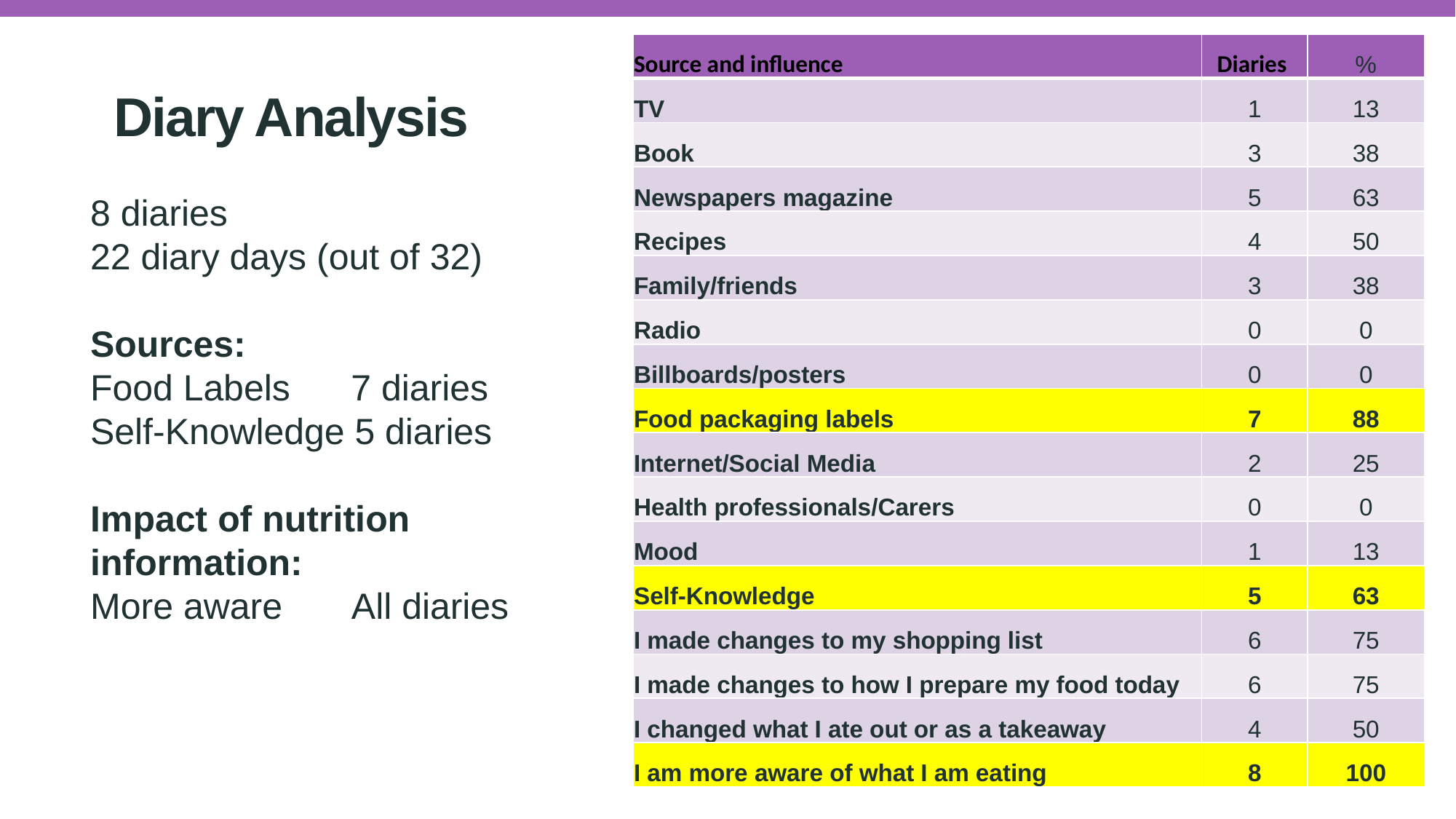

| Source and influence | Diaries | % |
| --- | --- | --- |
| TV | 1 | 13 |
| Book | 3 | 38 |
| Newspapers magazine | 5 | 63 |
| Recipes | 4 | 50 |
| Family/friends | 3 | 38 |
| Radio | 0 | 0 |
| Billboards/posters | 0 | 0 |
| Food packaging labels | 7 | 88 |
| Internet/Social Media | 2 | 25 |
| Health professionals/Carers | 0 | 0 |
| Mood | 1 | 13 |
| Self-Knowledge | 5 | 63 |
| I made changes to my shopping list | 6 | 75 |
| I made changes to how I prepare my food today | 6 | 75 |
| I changed what I ate out or as a takeaway | 4 | 50 |
| I am more aware of what I am eating | 8 | 100 |
# Diary Analysis
13
8 diaries
22 diary days (out of 32)
Sources:
Food Labels 7 diaries
Self-Knowledge 5 diaries
Impact of nutrition information:
More aware All diaries

## Slide 14
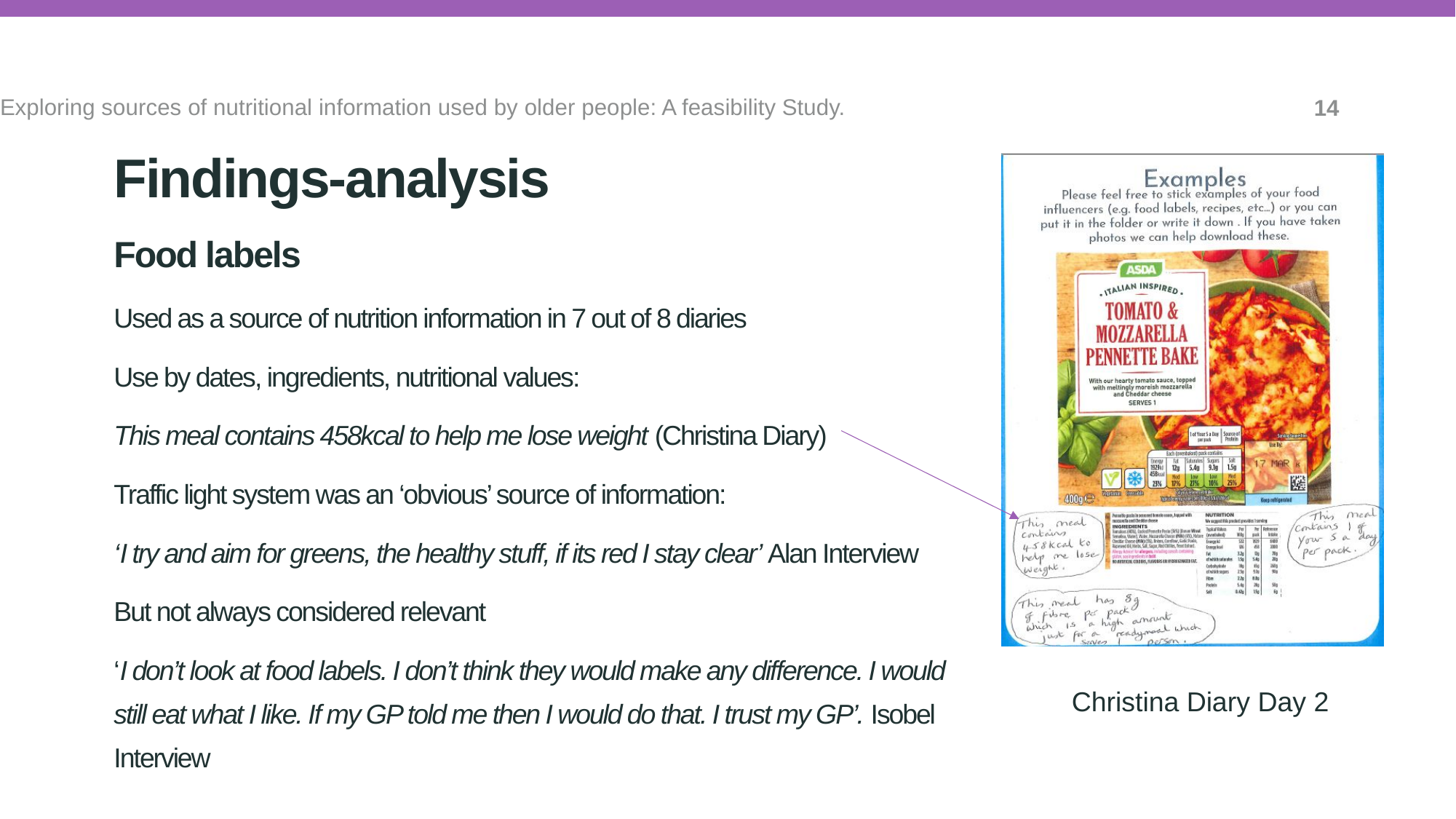

Exploring sources of nutritional information used by older people: A feasibility Study.
14
# Findings-analysis
Food labels
Used as a source of nutrition information in 7 out of 8 diaries
Use by dates, ingredients, nutritional values:
This meal contains 458kcal to help me lose weight (Christina Diary)
Traffic light system was an ‘obvious’ source of information:
‘I try and aim for greens, the healthy stuff, if its red I stay clear’ Alan Interview
But not always considered relevant
‘I don’t look at food labels. I don’t think they would make any difference. I would still eat what I like. If my GP told me then I would do that. I trust my GP’. Isobel Interview
Christina Diary Day 2

## Slide 15
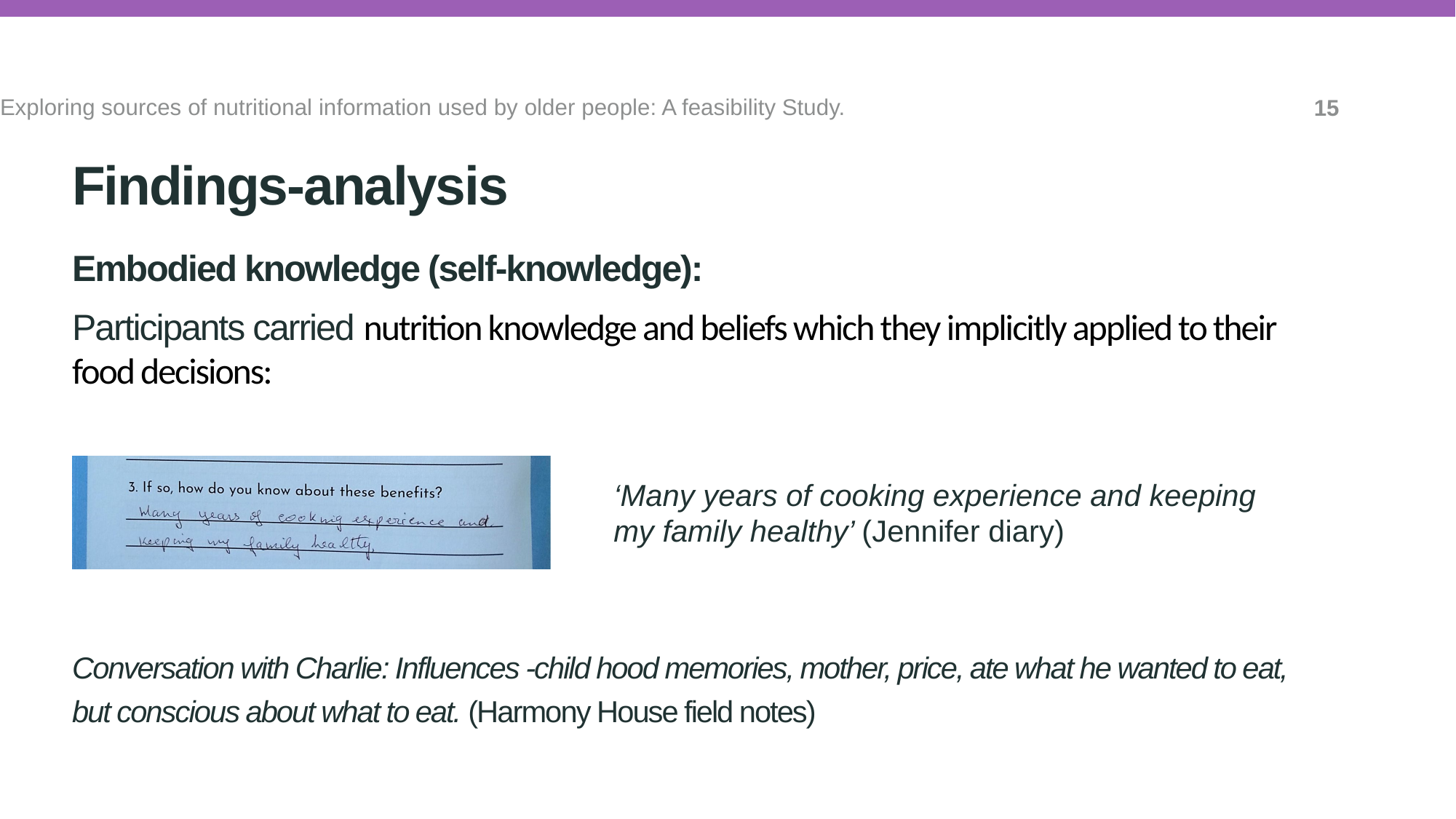

Exploring sources of nutritional information used by older people: A feasibility Study.
15
# Findings-analysis
Embodied knowledge (self-knowledge):
Participants carried nutrition knowledge and beliefs which they implicitly applied to their food decisions:
Conversation with Charlie: Influences -child hood memories, mother, price, ate what he wanted to eat, but conscious about what to eat. (Harmony House field notes)
‘Many years of cooking experience and keeping my family healthy’ (Jennifer diary)

## Slide 16
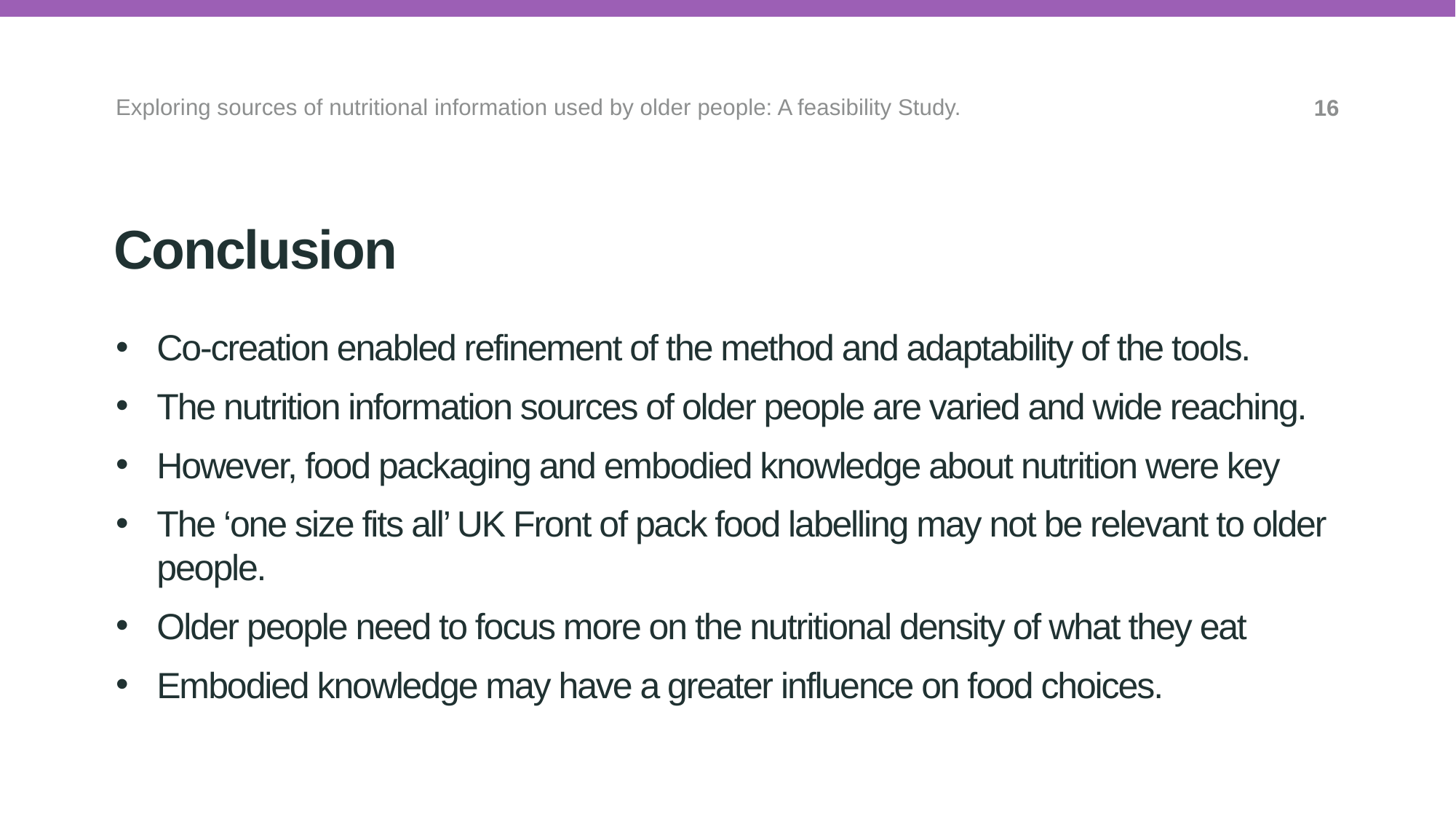

Exploring sources of nutritional information used by older people: A feasibility Study.
16
# Conclusion
Co-creation enabled refinement of the method and adaptability of the tools.
The nutrition information sources of older people are varied and wide reaching.
However, food packaging and embodied knowledge about nutrition were key
The ‘one size fits all’ UK Front of pack food labelling may not be relevant to older people.
Older people need to focus more on the nutritional density of what they eat
Embodied knowledge may have a greater influence on food choices.

## Slide 17
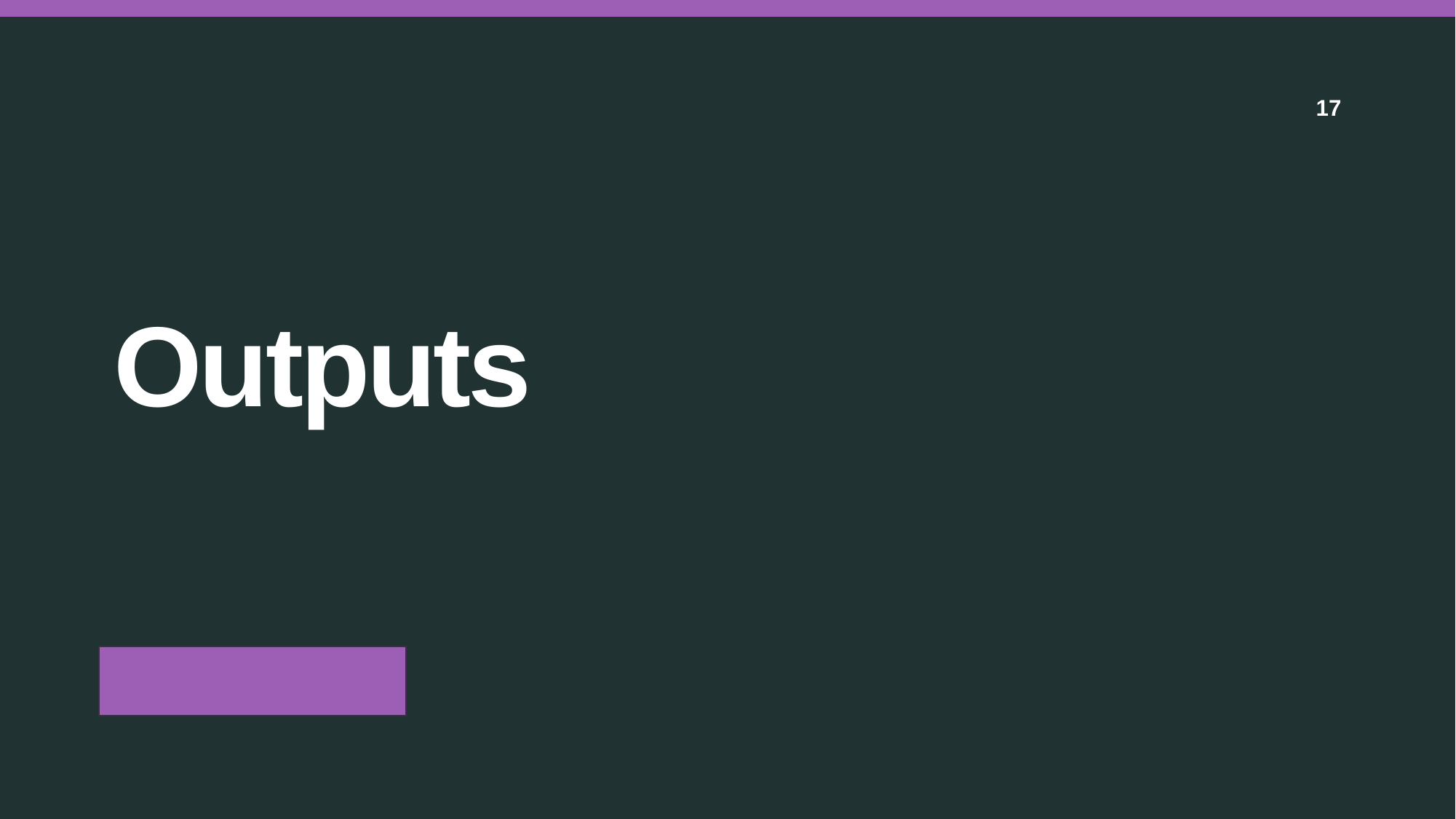

17
# Outputs

## Slide 18
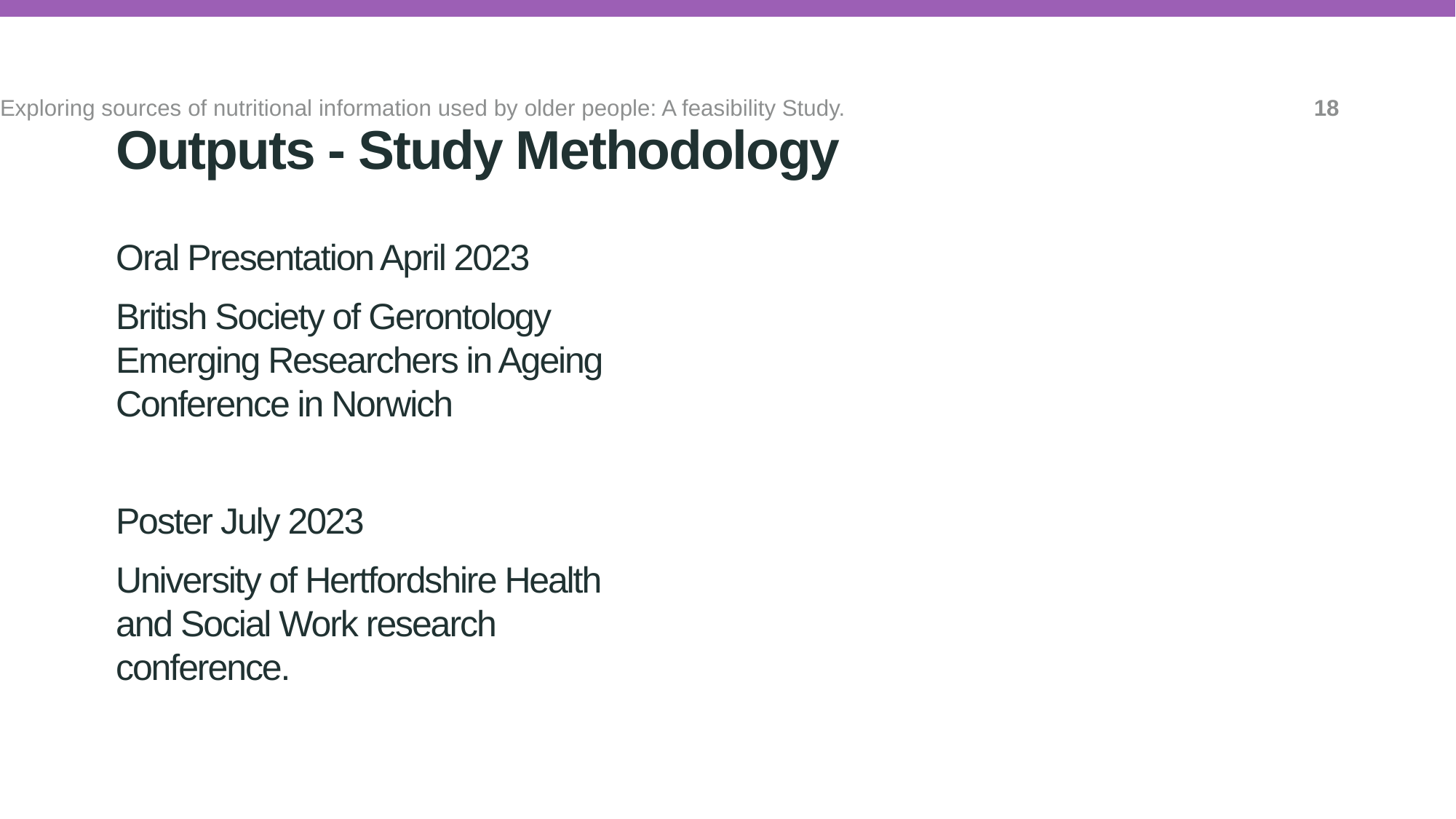

Exploring sources of nutritional information used by older people: A feasibility Study.
18
# Outputs - Study Methodology
Oral Presentation April 2023
British Society of Gerontology Emerging Researchers in Ageing Conference in Norwich
Poster July 2023
University of Hertfordshire Health and Social Work research conference.

## Slide 19
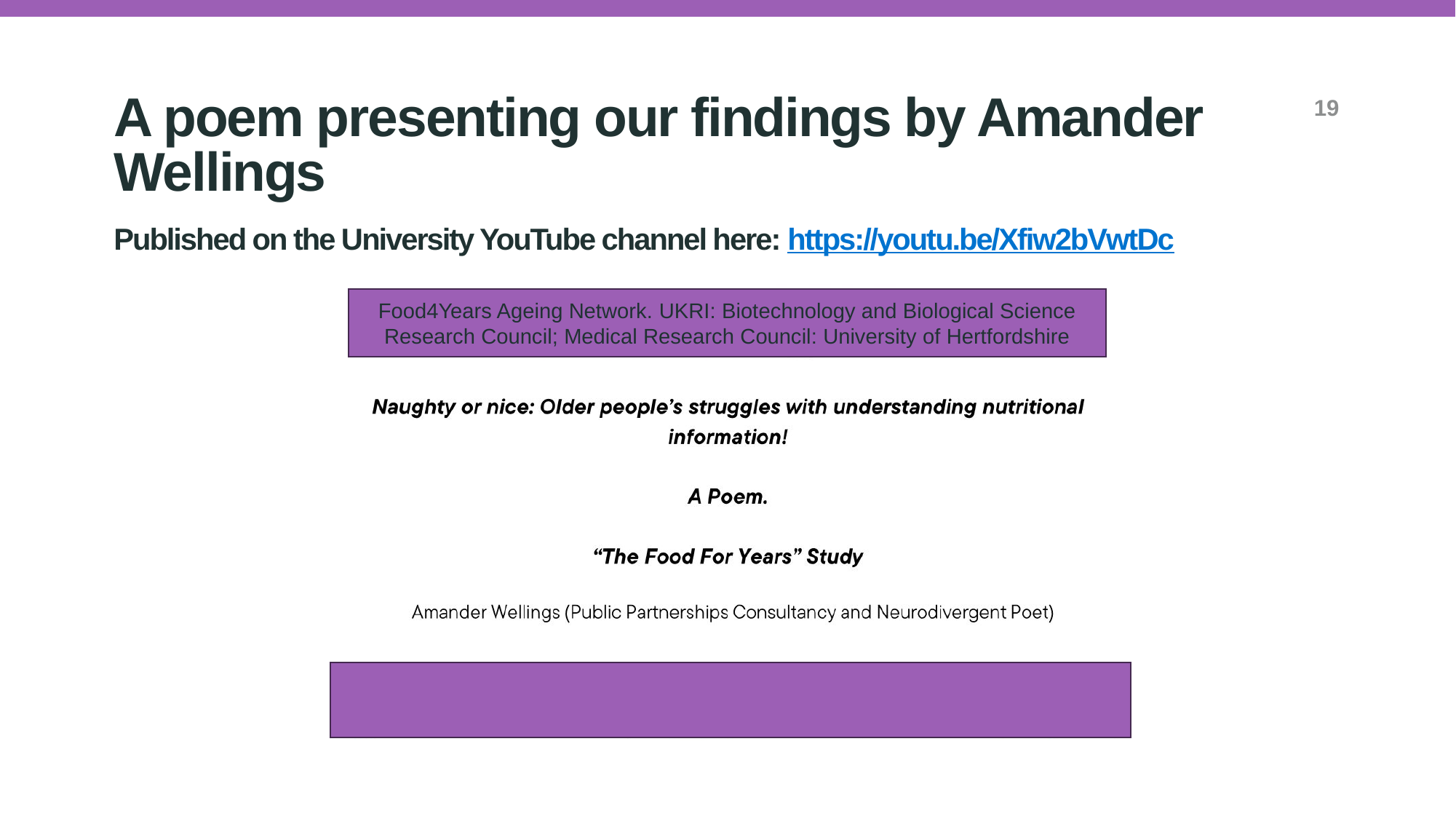

# A poem presenting our findings by Amander Wellings Published on the University YouTube channel here: https://youtu.be/Xfiw2bVwtDc
19
Food4Years Ageing Network. ​UKRI: Biotechnology and Biological Science Research Council; Medical Research Council: University of Hertfordshire​

## Slide 20
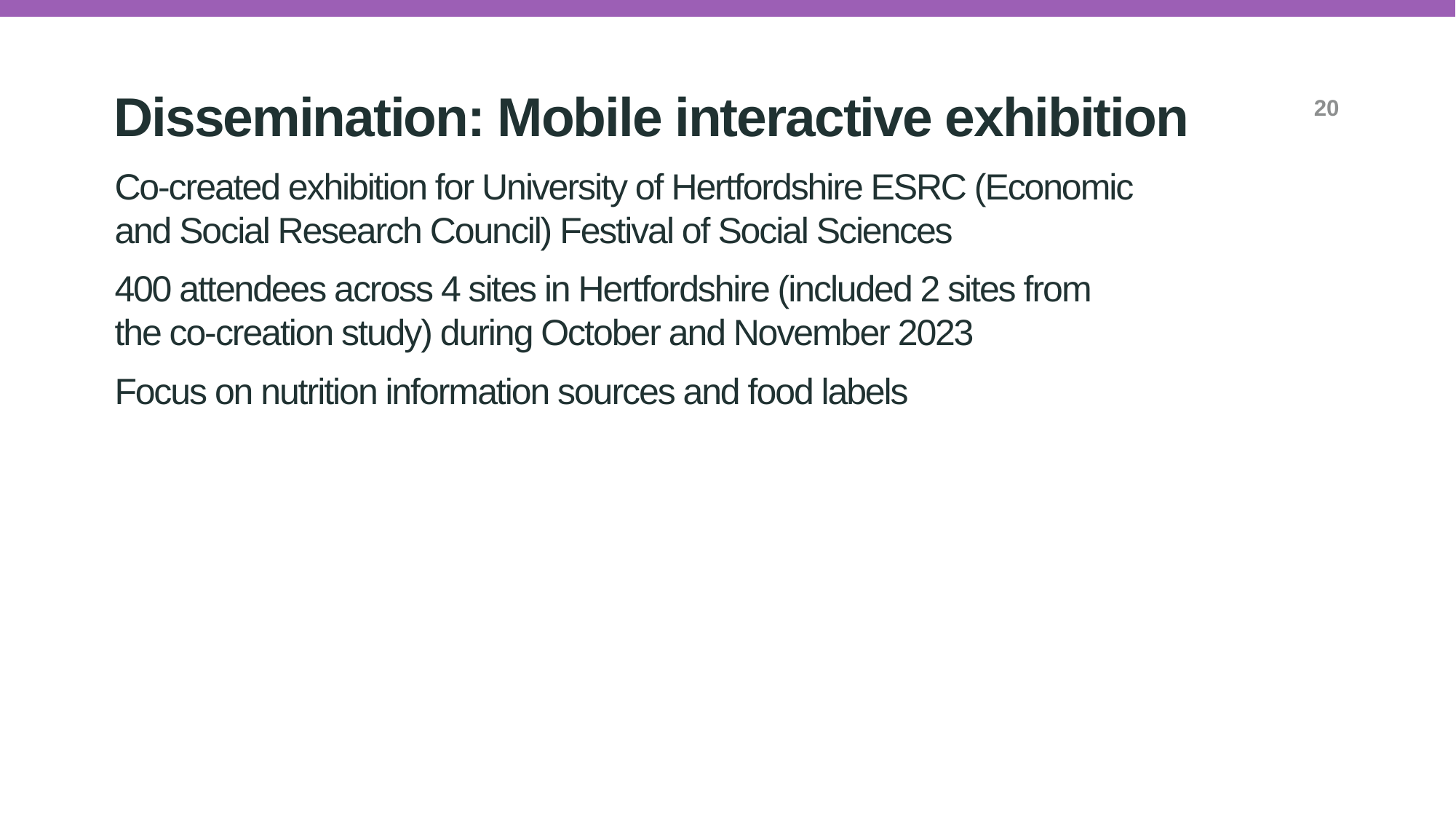

# Dissemination: Mobile interactive exhibition
20
Co-created exhibition for University of Hertfordshire ESRC (Economic and Social Research Council) Festival of Social Sciences
400 attendees across 4 sites in Hertfordshire (included 2 sites from the co-creation study) during October and November 2023
Focus on nutrition information sources and food labels

## Slide 21
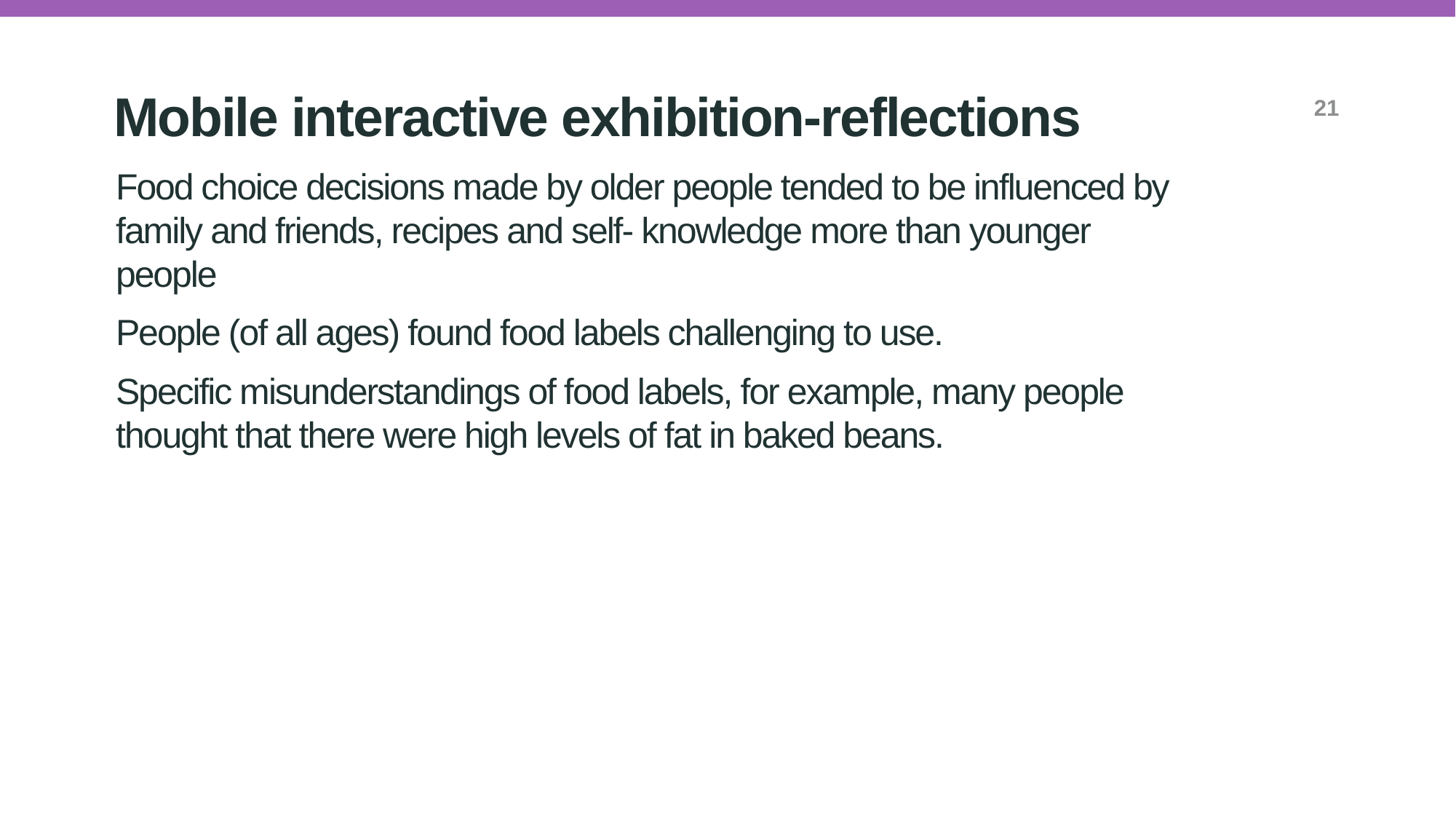

# Mobile interactive exhibition-reflections
21
Food choice decisions made by older people tended to be influenced by family and friends, recipes and self- knowledge more than younger people
People (of all ages) found food labels challenging to use.
Specific misunderstandings of food labels, for example, many people thought that there were high levels of fat in baked beans.

## Slide 22
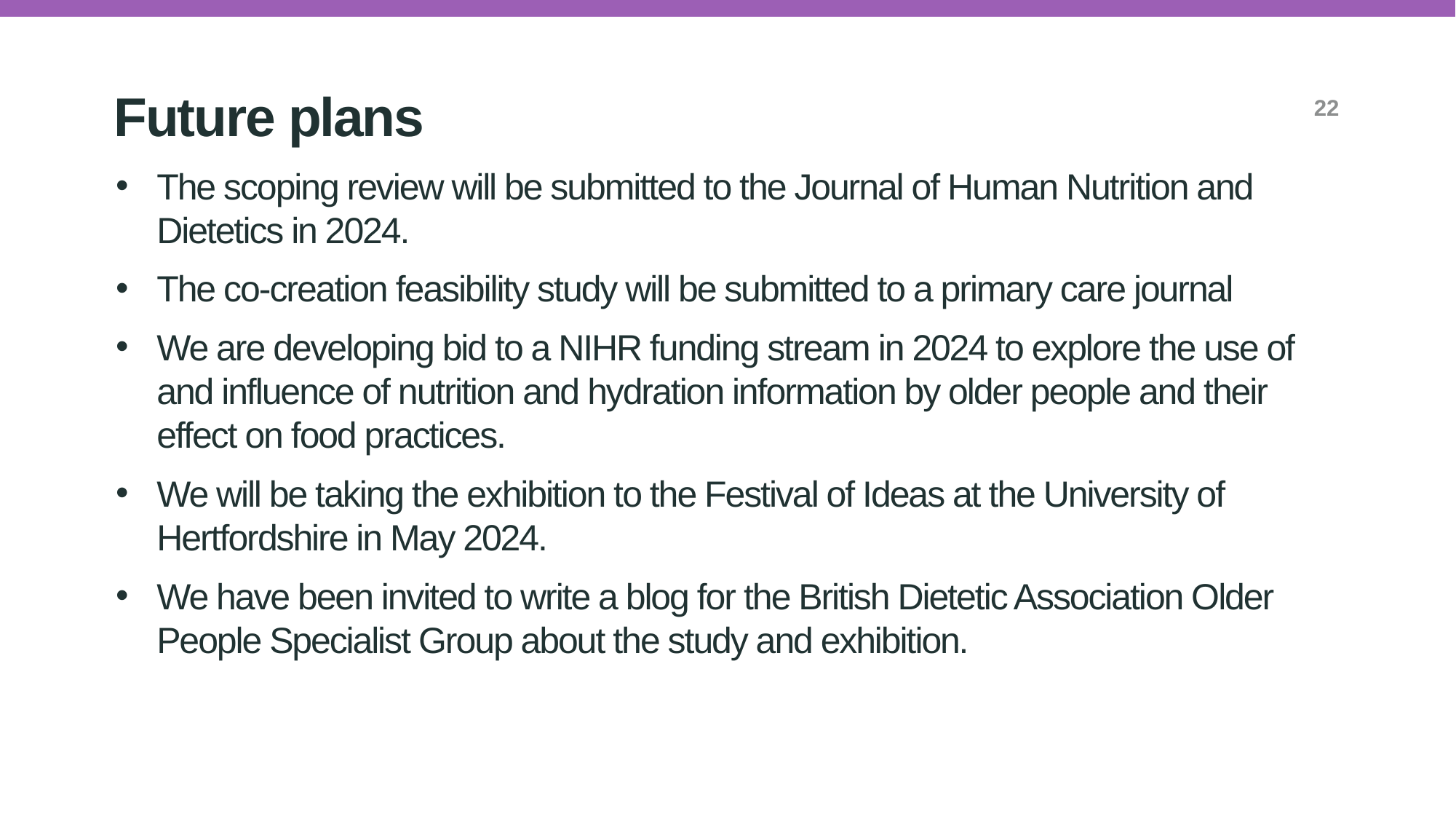

# Future plans
22
The scoping review will be submitted to the Journal of Human Nutrition and Dietetics in 2024.
The co-creation feasibility study will be submitted to a primary care journal
We are developing bid to a NIHR funding stream in 2024 to explore the use of and influence of nutrition and hydration information by older people and their effect on food practices.
We will be taking the exhibition to the Festival of Ideas at the University of Hertfordshire in May 2024.
We have been invited to write a blog for the British Dietetic Association Older People Specialist Group about the study and exhibition.

## Slide 23
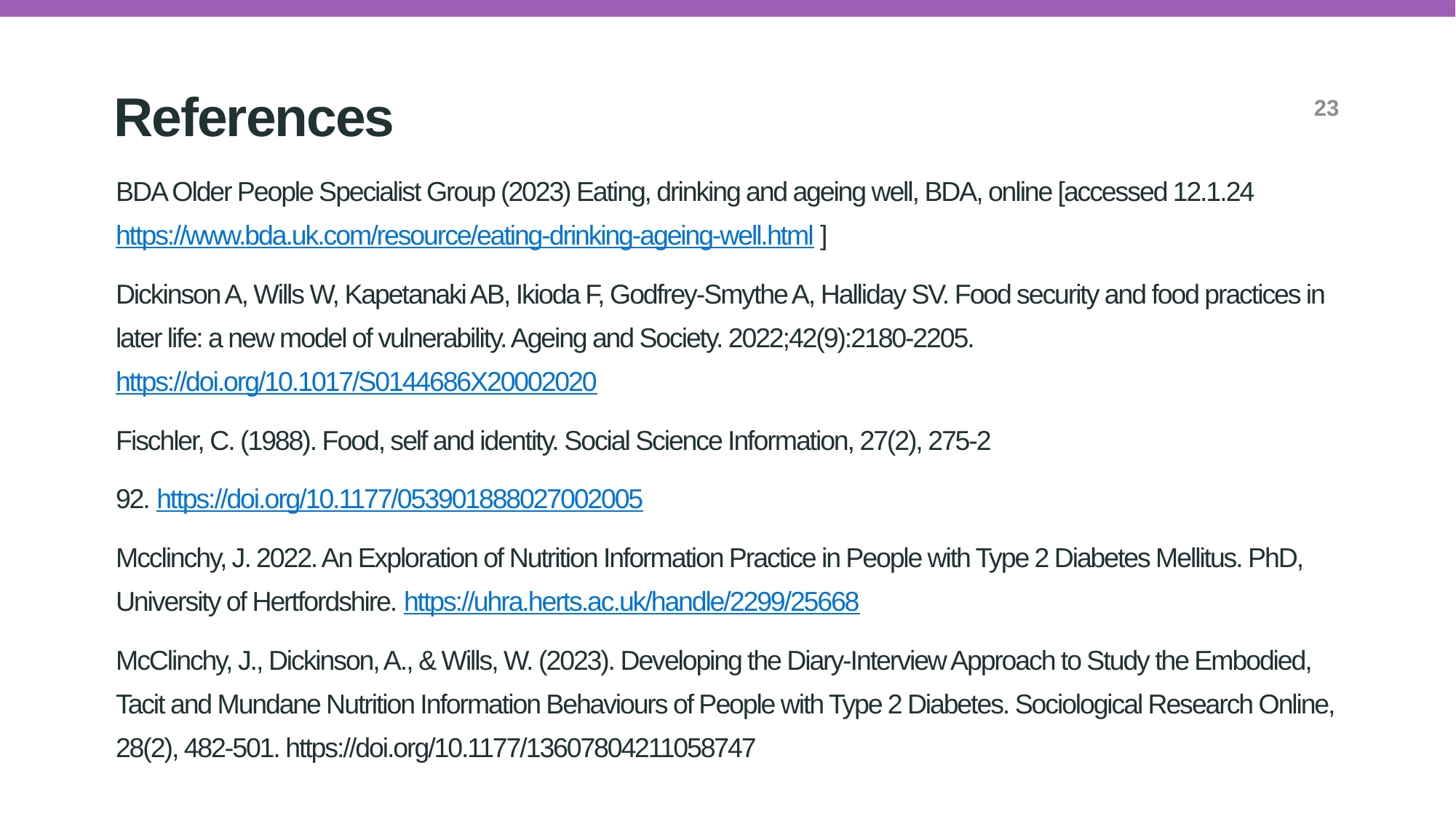

# References
23
BDA Older People Specialist Group (2023) Eating, drinking and ageing well, BDA, online [accessed 12.1.24 https://www.bda.uk.com/resource/eating-drinking-ageing-well.html ]
Dickinson A, Wills W, Kapetanaki AB, Ikioda F, Godfrey-Smythe A, Halliday SV. Food security and food practices in later life: a new model of vulnerability. Ageing and Society. 2022;42(9):2180-2205. https://doi.org/10.1017/S0144686X20002020
Fischler, C. (1988). Food, self and identity. Social Science Information, 27(2), 275-2
92. https://doi.org/10.1177/053901888027002005
Mcclinchy, J. 2022. An Exploration of Nutrition Information Practice in People with Type 2 Diabetes Mellitus. PhD, University of Hertfordshire. https://uhra.herts.ac.uk/handle/2299/25668
McClinchy, J., Dickinson, A., & Wills, W. (2023). Developing the Diary-Interview Approach to Study the Embodied, Tacit and Mundane Nutrition Information Behaviours of People with Type 2 Diabetes. Sociological Research Online, 28(2), 482-501. https://doi.org/10.1177/13607804211058747

## Slide 24
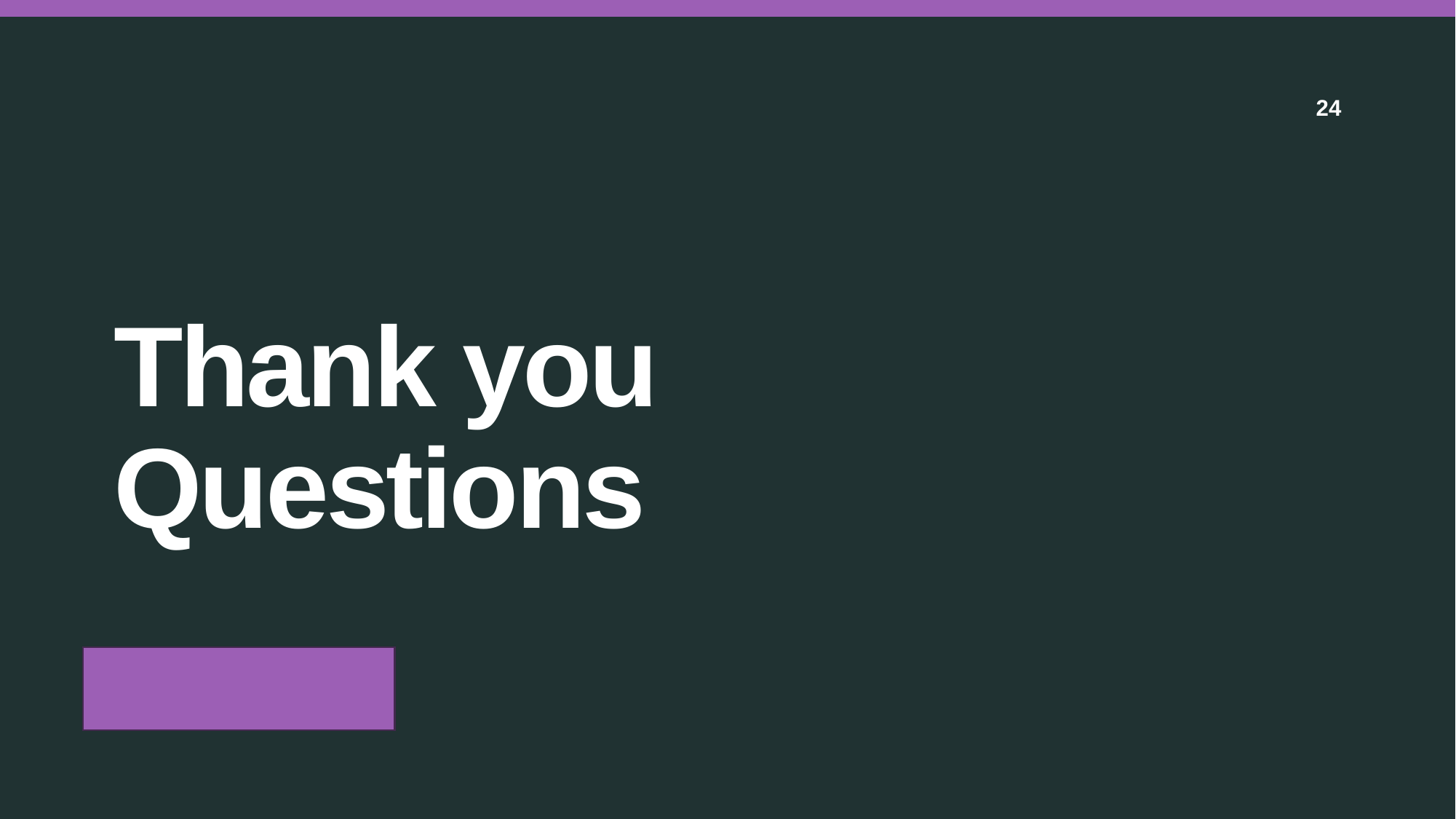

24
# Thank you Questions
