# Supplementary material for: Sources and preferences for nutrition information among older adults: A scoping review
Source: PLoS One. 2026 Feb 27;21(2):e0341015. doi: 10.1371/journal.pone.0341015 (PMC12948054; doi:10.1371/journal.pone.0341015)
Supplement: Attachment — Submitted filename: Peer Review_Sources of nutrition information.pdf [file pone.0341015.s005.pdf]

Sources of nutrition information used and preferred by older people: A scoping review

Peer Review Report

Title

The title can be rephrased, as the most suitable title for an academic journal should be based on clarity, scholarly tone, and alignment with scoping review conventions. My suggestion is:

"Sources and Preferences for Nutrition Information Among Older Adults: A Scoping Review"

This version is precise, avoids redundancy, and clearly signals the population, topic, and methodology. It uses "older adults," which is preferred in academic and public health literature over "older people," and it balances specificity with broad relevance for indexing and citation.

Abstract

The abstract clearly identifies the public health relevance of nutrition information for older adults, especially in the context of malnutrition risk. It outlines the scoping review methodology, including databases searched and inclusion criteria. The findings are succinctly presented, with attention to both source types and influencing factors. However, my suggestions are as follows:

1. **Redundancy:** Lines 14–16 repeat the aim unnecessarily. Consider condensing this into one clear sentence.

2. **Clarity of Findings:** The phrasing "8 studies showed…" and "6 studies showed…" is vague. Were these mutually exclusive? Did some studies report multiple sources?

3. **Terminology:** "Embodied knowledge" is mentioned without definition—this may confuse readers unfamiliar with the term.

4. **Impact and Implications:** The conclusion could more clearly articulate how future research or interventions might build on these findings.

Also, I suggested revising the Abstract. See the Suggested Revision of the Abstract below and you can add other information that are not included.

Revised Abstract -
A nutritionally adequate diet is essential for older adults to support healthy ageing and reduce the risk of malnutrition. With over a million older people in the UK affected or at risk, understanding where they source nutrition information is critical for designing effective public health interventions. This scoping review mapped existing studies on the sources and preferences for nutrition information among older adults. A comprehensive search of PUBMED, Scopus, and CINAHL (March 2023; updated February 2025) yielded 8936 records, of which 15 studies reporting on 14 research projects met inclusion criteria. Common sources included magazines, television, dietitians, general practitioners, family and friends, and personal experience ("embodied knowledge"), with educational level, gender, and trust influencing uptake and use. Further research is needed to assess the impact of these information sources and identify strategies to support older adults in making informed food choices that promote healthy ageing.

Introduction

1. Clarify the conceptual framework guiding the review—what theoretical lens informs the exploration of information sources?
2. Strengthen the rationale by linking the topic to broader public health goals such as SDG 2 (Zero Hunger) and SDG 3 (Good Health and Well-being).
3. Specify whether the review focuses on older adults in community settings, care homes, or both.
4. Consider integrating recent UK policy or demographic data to reinforce the urgency of addressing malnutrition in ageing populations.

Methods

1. Condense repetitive phrasing in some of the sub-sections and restate the aim and could be merged for clarity.
2. Provide more detail on inclusion/exclusion criteria—were qualitative studies included, and how was "preference" operationalized?
3. Clarify whether the review followed a formal framework (e.g., Arksey & O'Malley or PRISMA-ScR). Draw the PRISMA flow diagram again to reflect the Arksey & O'Malley PRISMA diagram.
4. Indicate whether any quality appraisal of included studies was conducted, even if not required for scoping reviews.

Results Presentation

1. Reorganize findings to distinguish between frequency of use and preference for sources—these are conceptually distinct.
2. Quantify overlaps—did some studies report multiple sources, and how were these counted?
3. Define “embodied knowledge” for clarity, as it may be unfamiliar to readers outside qualitative health research.
4. Consider presenting findings in a table or figure to improve accessibility and comparative insight.

Discussion

1. Deepen the analysis of how trust, gender, and education shape engagement with nutrition information.
2. Reflect on digital literacy and access—are older adults using online sources, and what barriers exist?
3. Compare findings with international literature to situate the UK context within broader ageing and nutrition trends.
4. Explore how cultural norms or generational attitudes may influence source preference and credibility.

Strengths and Limitations

1. Highlight the breadth of databases searched and the update in 2025 as strengths.
2. Acknowledge the limited number of included studies relative to the initial search yield.
3. Note potential publication bias—were grey literature or non-English studies excluded?
4. Discuss limitations in generalizability, especially if most studies focused on specific subgroups or regions.

Implications of Findings

1. Suggest how findings could inform tailored nutrition education strategies for older adults.
2. Recommend integrating trusted sources (e.g., GPs, family) into public health messaging campaigns.

3. Highlight the need for co-designed interventions that reflect older adults' lived experiences and preferences.
4. Encourage future research to explore the effectiveness of different information sources in changing dietary behaviour.

Conclusion/Recommendations

1. Reframe the conclusion to emphasize actionable insights for practitioners and policymakers.
2. Specify which stakeholders (e.g., NHS, local councils, charities) could implement the recommendations.
3. Suggest developing age-friendly nutrition communication tools that accommodate sensory, cognitive, and literacy needs.
4. Reinforce the importance of ongoing evaluation to assess the impact of information sources on dietary outcomes.

Proofreading and Editing

1. Eliminate redundancy in aim statements and streamline sentence flow.
2. Correct minor grammatical inconsistencies (e.g., "nutrition5 status" should be "nutrition status").
3. Ensure consistent terminology—use "older adults" or "older people" uniformly.
4. Improve transitions between sentences to enhance narrative cohesion.

References/In-Citations

1. Ensure all cited studies are current and relevant to the UK context or comparable ageing populations.
2. Include foundational scoping review methodology references (e.g., Levac et al., PRISMA-ScR).
3. Cite key public health reports on malnutrition in older adults (e.g., Age UK, NHS Digital).
4. Verify that all in-text citations match the reference list and follow the journal's formatting style.
